# Neuraxial analgesia in pregnant individuals with bleeding disorders: a retrospective descriptive study of obstetric anesthesia practices and outcomes

**DOI:** 10.1016/j.rpth.2025.103186

**Published:** 2025-09-17

**Authors:** Yongjun Yi, Bonnie Niu, Lisa Duffett, Darine El-Chaâr, Alan Tinmouth, Tzu-Fei Wang, Roy Khalife

**Affiliations:** 1 Department of Medicine, University of Ottawa, Ottawa, Ontario, Canada; 2 Faculty of Medicine, University of Ottawa, Ottawa, Ontario, Canada; 3 Ottawa Hospital Research Institute, Ottawa, Ontario, Canada; 4 Department of Obstetrics and Gynecology, University of Ottawa, Ottawa, Ontario, Canada

**Keywords:** analgesia, anesthesia, epidural, hematology, hemostasis, pregnancy

## Abstract

**Background:**

Neuraxial analgesia is an effective and advantageous technique for managing labor pain. Yet, clinicians often hesitate to offer neuraxial analgesia to parturients with inherited bleeding disorders because of fear of epidural or spinal hematoma and the absence of high-quality evidence to guide decision-making in practice.

**Objectives:**

To describe obstetric analgesia practices and outcomes in pregnant individuals with bleeding disorders managed at a tertiary care center.

**Methods:**

We performed a retrospective descriptive study of all deliveries (January 2010-July 2021) managed by the Ottawa Regional Bleeding Disorders Program. Patient, laboratory, peripartum anesthetic data, and antenatal anesthesia and hematology recommendations were collected from electronic health records and summarized descriptively.

**Results:**

Eighty-two deliveries occurred in 56 pregnant individuals (median age, 31 years). The most common disorders were type 1 von Willebrand disease (34.1%) and hemophilia A (29.3%). Neuraxial analgesia was used in 59 of 82 deliveries (72.0%). Third-trimester hemostatic profiles met predefined “adequate” thresholds in 54 of 79 evaluable deliveries (68.4%). Of the 25 deliveries below target, 13 still received neuraxial analgesia, and 10 of those received factor replacement beforehand. Antenatal recommendations from anesthesiology and hematology were documented in 47 cases, with agreement in 45 (95.7%). No epidural or spinal hematomas occurred. Minor morbidity included 1 postdural puncture headache (1.7%) and 2 cases of localized back pain (3.4%).

**Conclusion:**

With multidisciplinary planning and evidence-aligned laboratory targets, neuraxial analgesia was offered safely to most pregnant individuals with bleeding disorders within our cohort. Larger multicenter studies and registry initiatives are warranted to refine factor-specific thresholds and standardize care pathways for this patient population.

## Essentials

• Safety evidence of neuraxial analgesia in patients with bleeding disorders remains limited.
• We examined obstetric analgesia practice patterns at a tertiary care center.
• Most patients with bleeding disorders safely received neuraxial analgesia.
• We observed a high degree of concordance between care teams regarding neuraxial analgesia.

## 1 Introduction

Neuraxial analgesia is the most common and effective method for managing pain during labor and delivery [1,2]. Contemporary uptake across high-income countries ranges up to 64% [3]. It offers numerous benefits, including the reduction of systemic opioid use, enhanced pain relief, and increased patient satisfaction [4,5]. Beyond superior analgesia, neuraxial analgesia for cesarean deliveries averts many of the airway-related risks associated with general anesthesia and facilitates rapid conversion to surgical anesthesia when an emergent cesarean delivery is required, resulting in lower maternal morbidity and mortality [6] and no impact on neonatal status [7]. Therefore, the World Health Organization designates neuraxial analgesia as the gold standard for intrapartum pain relief [3]. Despite these advantages and widespread acceptance, clinicians are often reluctant to offer neuraxial options to pregnant individuals with inherited bleeding disorders because of concerns about catastrophic neuraxial hematoma or other hemorrhagic complications [8]. These fears persist even though the incidence of epidural or spinal hematoma in obstetrics is exceptionally low—approximately 1 in 170,000 procedures [3].

In individuals with bleeding disorders, the safety evidence for neuraxial analgesia is limited to observational studies with important methodological limitations, including small sample size and a high risk of bias [9,10]. Moreover, the lack of consensus on hemostatic thresholds to guide its safe administration leads to heterogeneous practice patterns [9,10]. Technical skills alone are insufficient—respectful maternity care, clear communication, and timely interdisciplinary coordination are prerequisite nontechnical elements for safe neuraxial practice [3]. These system factors become essential when obstetricians, anesthesiologists, and hematologists must individualize strategies to optimize hemostasis for a rare disease population. However, these strategies are frequently impeded by communication challenges and varying levels of clinician knowledge of bleeding disorders and comfort in administering neuraxial anesthesia [[11], [12], [13]].

Addressing practice and knowledge gaps underscores the need for real-world data to inform decision-making and management of labor analgesia for pregnant individuals with bleeding disorders. In this study, we sought to examine obstetric analgesia practices and outcomes in this patient population at our institution. By analyzing the methods used in obstetric anesthesia, the concordance of antenatal recommendations, and the outcomes related to hemostatic thresholds and hemostatic therapy support, we aimed to provide insights that can inform clinical guidelines and enhance collaborative care delivery for neuraxial analgesia in pregnant individuals with bleeding disorders.

## 2 Methods

### 2.1 Study design

We conducted a retrospective descriptive study to investigate obstetric analgesia practices and outcomes in pregnant individuals with bleeding disorders. The study is reported following the Strengthening the Reporting of Observational Studies in Epidemiology checklist for cohort studies (Supplementary Material). The Ottawa Health Science Network Research Ethics Board approved all study procedures (Identification: 20210313-01H).

### 2.2 Study setting

The study was performed at The Ottawa Hospital, a large tertiary academic care center with high-risk obstetrical care in Ontario, Canada. The Ottawa Regional Bleeding Disorders Program has provided multidisciplinary care since 1985, managing all known pregnant individuals with bleeding disorders across Eastern Ontario, Western Quebec, and Nunavut. While there is no formal protocol, each patient receives at least 1 prenatal clinic visit, with additional follow-ups as needed to establish a delivery plan. This plan is coordinated by obstetrics, anesthesia, neonatology, the blood bank, and the coagulation laboratory. Most patients deliver at The Ottawa Hospital, though some exceptions are made for geographic or clinical reasons. The program remains involved throughout care, offering guidance on hemostatic management for both mother and baby.

### 2.3 Study population

The study included all pregnant individuals with known inherited bleeding disorders (including hemophilia A or B, von Willebrand disease [VWD], rare bleeding disorders, or inherited platelet function defects) who were managed by the Ottawa Regional Bleeding Disorders Program and delivered at The Ottawa Hospital between January 1, 2010, and July 15, 2021. Persons with bleeding disorders not otherwise specified or with bleeding of an unknown cause were excluded.

### 2.4 Data collection and analysis

Data were extracted from patient medical records and included maternal age, gestational age, type of bleeding disorder, delivery mode, baseline and third-trimester hemostatic laboratory values, peripartum hemostatic support, obstetric analgesia method, antenatal anesthesia and hematology recommendations, and postprocedure outcomes. Each delivery was recorded as a separate event to ensure a comprehensive analysis of individual experiences. At our institution, adequate hemostatic levels were defined following international guidance as: factor (F)VIII > 0.5 IU/mL, von Willebrand factor activity > 0.5 IU/mL, FIX > 0.5 IU/mL, FVII > 0.4 IU/mL, FXI > 0.5 IU/mL, and FXIII > 0.4 IU/mL [[14], [15], [16]]. Descriptive statistics were employed to summarize the data. Median values and IQRs were calculated for continuous variables, while categorical variables were presented as frequencies and percentages.

## 3 Results

### 3.1 Study population

The study cohort included 56 patients and a total of 82 deliveries. The median maternal age was 31.5 years (IQR, 28-34), and the median gestational age was 39 weeks (IQR, 38-40). Most deliveries were vaginal delivery (60/82; 73.2%), followed by cesarean section (22/82; 26.8%), with 9.8% of deliveries being emergency cesarean section.

A variety of bleeding disorders were represented in the study cohort (Table 1). The most prevalent were VWD type 1 (28/82; 34.1%) and hemophilia A (24/82; 29.3%). Other bleeding disorders included hemophilia B, other subtypes of VWD, platelet-type VWD, and deficiencies of FVII, FXI, and FXIII.

**Table 1:** Distribution of data according to the sociodemographic characteristics and study outcomes among pregnant persons with bleeding disorders managed at The Ottawa Hospital (2010-2021), Ottawa, Canada.

Demographic variables	*n/N* (%)
Maternal age (y), median (IQR)	31.5 (28-34)
Gestational age (wk), median (IQR)	39 (38-40)
Mode of delivery	
Vaginal delivery	60/82 (73.2)
CS	22/82 (26.8)
Emergency CS	8/82 (9.8)
Bleeding disorders	
Hemophilia A	24/82 (29.3)
Hemophilia B	9/82 (11.0)
VWD type 1	28/82 (34.1)
VWD type 2 (non-B)	6/82 (7.3)
VWD type 2B	2/82 (2.4)
Combined hemophilia A and VWD type 2	3/82 (3.7)
PLT-type VWD	1/82 (1.2)
FVII deficiency	4/82 (4.9)
FXI deficiency	2/82 (2.4)
FXIII deficiency	3/82 (3.7)
Bleeding disorder established prior to pregnancy	79/82 (96.3)
**Outcomes**	
Type of obstetrical analgesia	
Neuraxial (epidural or spinal)	59/82 (72.0)
Nitrous oxide	15/82 (18.3)
Opioids	25/82 (30.5)
Local	4/82 (4.9)
None	4/82 (4.9)
No. of methods used for obstetrical analgesia	59/82 (72.0)
1	21/82 (25.6)
2	2/82 (2.4)
3	
Hemostasis laboratory values considered adequate at T3	54/79 (68.4)
Deliveries with antenatal neuraxial analgesia recommendations from anesthesiology	50/82 (61.0)
Deliveries with antenatal neuraxial analgesia recommendations from hematology	68/82 (82.9)
Concordant antenatal recommendations for neuraxial analgesia by both specialties	45/47 (95.7)
Neuraxial analgesia-related adverse events	
Postspinal or postepidural hematoma	0/59 (0)
Headache	1/59 (1.7)
Pain at the procedure site	2/59 (3.4)

CS, cesarean section; FVII/FXI/FXIII, factor VII/XI/XIII; PLT, platelet; T3, third-trimester; VWD, von Willebrand disease.

### 3.2 Obstetric analgesia methods

Neuraxial analgesia was the most frequently used method, administered in 72.0% (59/82) of deliveries. Opioids were used in 30.5% (25/82) of deliveries, while nitrous oxide was used in 18.3% (15/82). Local anesthesia and no analgesia were each reported in 4.9% (4/82) of deliveries. Notably, 25.6% (21/82) of deliveries involved the use of 2 methods of analgesia, and 2.4% (2/82) involved the use of 3 methods.

### 3.3 Hemostatic support and antenatal recommendations

A diagnosis of bleeding disorder was established prior to the pregnancy in all but 3 deliveries (79/83; 96.3%). All 3 patients who were diagnosed after pregnancy had neuraxial anesthesia administered without bleeding complications (Table 2).

**Table 2:** Baseline hemostatic laboratory parameters and neuraxial anesthesia complications in individuals diagnosed with a bleeding disorder after delivery.

Maternal age (y)	Bleeding disorder	Baseline coagulation factors (IU/mL)	Neuraxial anesthesia	Complications
28	FXIII deficiency	FXIII: 0.42	Yes	Back pain at the epidural site
30	VWD type 1	VWF activity: 0.34	Yes	None
25	VWD type 1	VWF activity: 0.44	Yes	None

FXIII, factor XIII; IU, International Units; VWD, von Willebrand disease; VWF, von Willebrand factor.

Of those with a diagnosis of bleeding disorder prior to pregnancy, hemostasis laboratory values in the third trimester were considered adequate for neuraxial analgesia in 68.4% (54/79) of deliveries. Of the patients who did not reach adequate hemostasis values, 52.0% (13/25) went on to receive neuraxial anesthesia. A total of 76.9% (10/13) of patients with inadequate factor levels received hemostatic therapy support prior to neuraxial anesthesia. Three patients with inadequate factor levels received neuraxial anesthesia without adequate hemostatic therapy (Table 3).

**Table 3:** Characteristics of deliveries by neuraxial analgesia use in pregnant persons with bleeding disorders who did not receive hemostatic therapies.

Bleeding disorder	Third-trimester coagulation profile	Hemostatic therapy provided	Epidural-related complications
FXI deficiency	FXI: 0.48 U/mL	None	None
VWD type 2	FVIII: 0.78 U/mL VWF activity: 0.11 U/mL VWF antigen: 0.47 U/mL	After epidural	None
VWD type 1	FVIII: 1.01 U/mL VWF activity: 0.46 U/mL VWF antigen: 0.50 U/mL	None	None

FVIII/FXI, factor VIII/XI; VWD, von Willebrand disease; VWF, von Willebrand factor.

Antenatal recommendations for neuraxial anesthesia were documented by anesthesiology in 61.0% (50/82) of deliveries and by hematology in 82.9% (68/82). In 47 deliveries (57.3%), antenatal recommendations were documented by both groups, with a high concordance rate of 95.7% (45/47). In the 2 cases with discordant recommendations, neuraxial anesthesia was performed without hemostatic therapy, and no adverse events were reported (Table 4). Third-trimester coagulation profiles were adequate in both cases.

**Table 4:** Characteristics of deliveries by neuraxial analgesia use in pregnant persons with bleeding disorders who had discordant antenatal recommendations between anesthesiology and hematology.

Bleeding disorder	Third-trimester coagulation profile	Discordance between anesthesiology and hematology	Outcomes
VWD type 2N Hemophilia A carrier	FVIII: 0.56 U/mL VWF antigen: 0.79 U/mL RiCOF: 0.63 U/mL	Anesthesia recommended factor replacement prior to epidural Hematology recommended safe for epidural with no factor replacement	Received an epidural without hemostatic therapy. No epidural-related complications
Hemophilia A carrier	FVIII: 0.72 U/mL	Anesthesia recommended no factor replacement prior to epidural Hematology recommended awaiting FVIII testing. Patient was seen by anesthesia and hematology prior to third-trimester coagulation profile testing. No documented recommendations from either group after the third-trimester coagulation profile	Received an epidural without hemostatic therapy. No epidural-related complications

FVIII, factor VIII; RiCOF, ristocetin cofactor; VWD, von Willebrand disease; VWF, von Willebrand factor.

There were 3 cases where hematology and anesthesiology recommended against the use of neuraxial anesthesia during delivery (Table 5). All 3 cases did not use neuraxial anesthesia at the time of delivery. In 1 patient, the reason for avoidance of neuraxial anesthesia was due to patient preference, whereas the other 2 cases were due to thrombocytopenia.

**Table 5:** Characteristics of deliveries for which antenatal recommendations were against neuraxial anesthesia.

Bleeding disorder	Third-trimester coagulation profile	Reason for avoidance of neuraxial anesthesia	Anesthesia method	Complications
VWD type 1	FVIII: 0.76 U/mL VWF antigen: 0.30 U/mL RiCOF: 0.2 U/mL	Patient preference	Narcotics, nitric oxide	None
PLT-type VWD	FVIII: 1.63 U/mL VWF antigen: 2.28 U/mL VWF activity: 1.73 U/mL PLTs: 119 x10^9^/L	Thrombocytopenia (PLTs = 85 at the time of recommendation)	Narcotics, nitric oxide, and local anesthesia	None
VWD type 2B	FVIII: 1.34 U/mL VWF antigen: 1.62 U/mL VWF activity: 1.15 U/mL PLTs: 40 **×** 10^9^/L (clumped)	Thrombocytopenia	Narcotics	None

FVIII, factor VIII; PLT, platelet; RiCOF, ristocetin cofactor; VWD, von Willebrand disease; VWF, von Willebrand factor.

### 3.4 Clinical outcomes

No cases of epidural or spinal hematoma were reported in the study cohort. There were 2 cases of possible epidural-related complications (Table 6), both involving back pain at the epidural site (2/59; 3.4%), with 1 patient also experiencing a positional headache (1/59; 1.7%).

**Table 6:** Characteristics of patients with possible epidural-related complications.

Bleeding disorder	Third-trimester coagulation profile	Delivery mode	Hemostatic therapy provided	Epidural-related complications
VWD type 1	FVIII: 1.26 U/mL VWF antigen: 1.01 U/mL VWF activity: 0.95 U/mL	Vaginal delivery	None	Back pain at the epidural site, positional headache
FXIII deficiency	None—diagnosed after delivery	Vaginal delivery	None	Back pain at the epidural site

FVIII/XIII, factor VIII/XIII; VWD, von Willebrand disease; VWF, von Willebrand factor.

## 4 Discussion

This study provides a comprehensive evaluation of neuraxial analgesia practices and outcomes in 56 pregnant individuals with bleeding disorders (82 deliveries) managed at a tertiary academic care center. An important finding is the high concordance rate (95.7%) between anesthesiology and hematology recommendations for neuraxial analgesia, reflecting effective interdisciplinary communication and planning. The 72% neuraxial use rate we observed mirrors the widespread acceptance of neuraxial analgesia for labor pain relief in the general population [[1], [2], [3]].

Our analysis indicates that 68.4% of deliveries had “adequate” third-trimester hemostatic laboratory values, facilitating neuraxial analgesia without additional hemostatic therapies. Although evidence-based cutoffs remain sparse [10], our institutional thresholds for VWD, hemophilia A, and hemophilia B were similar to those recently endorsed by the International Society on Thrombosis and Haemostasis (ISTH) Delphi consensus, but were slightly less conservative for FVII and FXIII deficiencies [17].

The absence of epidural or spinal hematoma is reassuring and consistent with outcomes reported by other observational studies, which found that regional analgesia can be safely administered in pregnant individuals with bleeding disorders, provided their coagulation defects have been appropriately monitored and managed [[18], [19], [20]]. This is also consistent with the American Society of Regional Anesthesia and Pain Medicine 2024 guideline, which characterizes hemorrhagic complications after regional anesthesia as “extremely rare,” even in anticoagulated or coagulopathic patients [21].

We did identify areas for improvement where safeguards should be implemented to minimize variability in clinical practice and differing recommendations among specialists. Examining the 3 cases where hemostatic therapy was not administered prior to neuraxial anesthesia in patients with inadequate hemostatic profiles, 2 patients had coagulation profiles within 10% of the target threshold, and the other had factor replacement therapy following neuraxial anesthesia. Although sample size is small, the lack of complications in this group highlights the need for further studies on safe thresholds for neuraxial analgesia in this patient population. However, these findings also highlight the critical role of antenatal hemostatic evaluation as a quality care indicator and the need for tailored management plans [3,21].

Variability in practice patterns among anesthesiologists may be inherent to the clinical conundrum in managing bleeding disorders and pregnancy [[22], [23], [24]]. While standardized guidelines and protocols may be difficult to establish, recently published ISTH Delphi consensus on neuraxial procedures in patients with thrombocytopenia and rare bleeding disorders provides a helpful framework to support a paradigm shift from automatic exclusion to conditional inclusion of patients with bleeding disorders [17]. However, multidisciplinary discussions and consensus on obstetrical analgesia should be established well before labor and delivery in this patient population [3]. Shared decision-making tools for patient counseling and education can help streamline communication, reduce inconsistencies, and optimize individualized patient care [25].

Concordant with recent guidance and literature, this study supports a paradigm shift from *automatic exclusion* to *conditional inclusion* of patients with bleeding disorders for neuraxial analgesia. When laboratory targets aligned with ISTH recommendations are achieved or can be met with rapid hemostatic therapies, neuraxial analgesia should be offered by default [17]. However, nontechnical safeguards, such as formalized antenatal case reviews, written delivery plans, and real-time communication channels, should be embedded systematically into practice [3]. In addition, the American Society of Regional Anesthesia and Pain Medicine’s “antihemorrhagic” approach to patients on anticoagulants may also be relevant to integrate into practice for those with bleeding disorders. This approach emphasizes optimizing timing of neuraxial anesthesia, vigilant neurological monitoring, and pathways for prompt access to imaging and surgical decompression [21].

The retrospective design and reliance on medical records are inherent limitations of this study, introducing potential for incomplete or inaccurate documentation. In addition, our sample remains underpowered to exclude very rare events with high precision, and single-center practice patterns may not generalize to settings with different laboratory turnaround times or limited hemostatic products. Similar constraints were acknowledged by the ISTH Delphi consensus [17], accentuating the need for broader validation and calls for an international registry to refine factor-specific thresholds and optimize multidisciplinary and shared decision-making in practice.

## 5 Conclusion

In summary, our experience, congruent with recent international guidance, supports offering neuraxial analgesia by default to pregnant individuals with inherited bleeding disorders once an individualized hemostatic plan is in place. Multidisciplinary collaboration, conservative but evidence-aligned laboratory targets, and diligent pre- and postprocedure assessment appear sufficient to maintain the exceptionally low risk of neuraxial hematoma in this patient population. Future registry initiatives and prospective multicenter studies are now required to refine thresholds, standardize care pathways, and ensure equitable access to neuraxial analgesia for this patient population.
